# 

**Published:** 1888-03-10

**Authors:** 


### SPECIAL NOTICE TO NEWSAGENTS, ADVERTISERS, AND THE TRADE.

### CHANGE OF OFFICE AND PUBLISHER.

Notice is hereby given that the Office of The Hospital will henceforth be at 38, Old Jewry (Cheapside end), E.C., to which address all communications should be sent. Mr. A. Doig has been appointed Advertising Agent, and Mr. J. H. S. Hanning, Manager. All Cheques and Post Office Orders should be made payable to J. H. S. Hanning, 38, Old Jewry, E.C. crossed Lloyds, Barnetts, and Bosanquets Bank (Limited).

### NURSING MIRROR.

Several communications for the "Mirror," the "Editor's Letter Box." and also "Amusements for Convalescents" are unavoidably held over until next week in consequence of the great pressure on our space.

### Amusements with Profit for All Ages.

**Rules.**—All answers must be addressed to the Prize Editor, The Hospital, 38, Old Jewry, Cheapside, London, E.C., not later than the *first* post on Thursday next. The full name and address must in all cases be given, but a *nom de plume* may be added for publication. Only one side of the paper must be written on.

### FOR MEN AND WOMEN.

**Rule.**—Competitors can enter for *all* quarterly competitions, but no competitor may take more than *two quarterly prizes* during the year.

### Acrostic Competition.

Second quarter commenced February 25th, 1888; ends May 19th, 1888.

A PRIZE of ONE GUINEA will be given to the competitor who gains the highest number of marks for best original acrostics during the ensuing quarter. All acrostics sent in for this competition will be credited according to merit, twelve marks being the highest score given for one acrostic.

A PRIZE of HALF-A-GUINEA to be given to the competitor who gains the highest score for solution of acrostics set. *One mark* only will be given to the competitors who solve all the lights of each acrostic.

Exception will be made in the case of *quotation acrostics*, when *two marks* will be given, *one* for the correct solution of all *lights*, and *one* mark for the *source* of the quotations.

This week credit will be given for solution of

No. 2. Double Acrostic.

To some who live in these enlightened days,
My *primals* seem the worthier of praise;
But we, who faithful are to Queen and land,
Declare my *finals* shall securely stand.

1. If rightly used I pleasure give to old and young,
Without my aid no tales of love nor war were sung.
2. *Mine* the reflection ot't of merry joy or hopeless woe;
My voice now far, now near, deceives with words now loud, now low.
3. In *me* lies latent power to help all human ill or good,
In ready hands, like living things to make thought understood.
4. Welcome in summer heat or wintry rain I may appear,
Companion strong of daily walk for many a year.
5. Beautiful in life and death, God of the summer sun!
Mourned by the Gods themselves when thy sweet life by fraud was done.
6. One of spring's lovely messengers to earth, pale, fragrant, sweet,
Harbinger of further joys, when summer's balmy breath we meet
7. Though small myself, *my* ttle aid makes large things larger still,
As every drop of dew or rain helps form the flowing rill.
8. Last but not least, on virtues roll *my* radiance doth appear,
Halo of heavenly love, brightening earth's darkness drear.

### Answers.

No. 1. Double Acrostic.

C ame O
H amme R
A hme D
O mphal E
S aty R

Correct answers have been received from Brownie, Dunce, Gretta, Ostrich, Mater, E. E., Reldas, Lady Clara, Rowdy Corner.

### (A) THE WORD COMPETITION.

Second Quarterly Competition commenced February 4th, 1888; ends April 28th, 1888.

Three prizes of one guinea, 15*s*. and 10*s*. 6*d*. will be given each quarter to the three competitors who obtain the highest number of marks: (1) by making the largest number of words derived from the word or phrase set for anatomical dissection; or (2) for the best answers to (B); or (3) by (1) and (2) combined.

We shall give the newspapers and periodicals or dissection during this quarter, that for this, the *sixth* week of the quarter, being

"Guardian."

Observe.—Proper names, abbreviations, foreign words, words of less than four letters, and repetitions are barred; plurals, past and present participles, of verbs are allowed. Nuttall's *Standard* dictionary only to be used.

N.B.—All words sent in must be arranged *alphabetically*, with correct total affixed.

### Results of Fourth Week.

*Academy.*—Dollie (17), Ellice (17), Patience (15), Gretta (14), Lauriston (14), Nelle (14), E. E. (13), Lightowlers (13), Lodge (13), Isabel (13), Condorcet (12), Brisbane (12), Butterfly (12), Lancashire Witch (12), Sym (12), Dunce (11).

### (B) THE LITERARY COMPETITION.

Some people hate puzzles of all kinds, including word competitions, but they are fond of correspondence, and many sisters and mothers are continually receiving and sending letters to and from members of the family who are abroad in India or the Colonies. The constant coming and going between England and other portions of the British Empire make it a matter of interest and importance to know something about the life, the climate, the amusements, the adventures, the clothing, and the surroundings of those who seek a livelihood beyond the seas. We shall therefore try to find space for the publication of selected letters from abroad, which may be sent by any of our readers who have friends and relations in foreign lands. We shall give marks for these letters as well as for the word competitions which will count equally for these prizes, so that anyone may enter for both or either, the prizes to be given to those who get the highest number of marks.

### Notices to Correspondents.

*Lightowlers* has forwarded a letter from the Cape for insertion in this column. We shall be glad if she will send a brief explanation of the work to which reference is made. We are still open to receive letters of interest from abroad.

*Plummy Fluff.*—Yes, certainly.

### A NEW P. AND O. STEAMSHIP.

(*From a Correspondent.*)

On Thursday the largest steamship which has ever been constructed in Ireland—a vessel 466 ft. in length, and of 6,380 tons gross register—was handed over by her builders, Messrs. Harland and Wolff, of Belfast, to the owners, the Peninsular and Oriental Steam Navigation Company. Her triple expansion engines indicate some 7,000-horse power, and she will be able to maintain 16-knots at sea. It will be seen, therefore, that the Admiralty knew what they were about when they selected her as one of the fast armed cruisers which they have subsidized the P. and O. Company to hold at the command of Government. The *Oceana* has been fitted under the superintendence of the Chief Constructor of the Navy with gun racers, and other appliances, so that she could be equipped as a man-of-war on the shortest notice. She could carry 2,500 troops with ease.

It is, however, in the interests of peace and commerce that her main work is to be carried on, and she is destined to play an important part in the carrying trade between this country and the Australian colonies. She leaves London on the 9th March with the colonial mails for Adelaide, and will proceed thence to Melbourne and Sydney filled with a large number of first and second-class passengers, for whose comfort luxurious provision has been made. Her dining saloons, her drawing-room, and smoking-rooms, in light oak and in walnut, have all been designed by Mr. Colcutt, the architect of the Imperial Institute, and these, with the sleeping cabins, roomy and well ventilated, fitted with folding lavatories, with spring mattresses and chests of drawers, leave nothing to be desired. The creature comforts of the passengers have been well provided for by the refrigerating apparatus, which will not only provide the 250 first-class and 160 second-class passengers which she will carry with every imaginable dainty, but will serve to carry home frozen meat from the colonies for the English market if occasion arises.

Messrs. Harland and Wolff have a sister ship nearly completed for the same company named the *Arcadia*, to be employed in the same trade; and these, with the two equally large armed cruisers built at Greenock by Messrs. Caird and Co., the *Victoria* and *Britannia*, will form four of the finest vessels in this great company's large fleet.

### THE CHILDREN'S CORNER.

PRIZES.
FOR MOST CORRECT ANSWERS TO PUZZLES.
This Commenced October 1st, 1887.
One Pound a Month.

A PRIZE OF THREE GUINEAS to the Competitor who shall have sent in the largest number of correct or best answers during the twelve months.

### Puzzles—Second Week.

No. 5. Riddle-me-ree.

My *first* is in *dear*, but not in *cheap*.
My *second* is in *wake*, but not in *sleep*.
My *third* is in *chair*, but not in *stool*.
My *fourth* is in *chest*, but not in *tool*.
My *fifth* is in *hay*, but not in *mow*.
My *whole* is a *flower* which all of you know.

No. 6. Historical Acrostic. Initals and finals form the name of a captive king and his brave deliverer. 1. A medicinal root. 2. Pastoral poem. 3. Company. 4. Used on a coach. 5. Held fast. 6. Found in a fish. 7. Quaint and amusing.

No. 7. Charade. My *second* comes out in the sun, and my *first* disappears in it, my *whole* cannot exist without it.

No. 8. Literary Conundrum. My *first* feels jolly, my *second* is not old, my *third* is best with mint sauce, and my *whole* splits up into three separate words, meaning two poets and a great essayist.

### Results of Fourth Week Puzzles.

No. 13. Geographical Acrostic.

A de N
L lanell Y
B ahi A
E hrenbreitstei N
R odrigue Z
T arragon A

No 14. Cap.
No. 15. Charade.—Bank Note=Banknote.
No. 16. Enigma.—Silence.

*All right*—A. C. K., Excelsior, A. G. S., McCulloch, Plummy Fluff, Mount Edgcumbe, Alert, Oswald, H. A. T. *Three right*—Alsie, Gem, Spider, Jerusalem, Scrooge, Robinson. *Two right*— Judy, Happy Eliza, Moina.

### Results of Fifth Monthly Competition.

*Oswald H*, *Alert*, and *Excelsior* having each gained the highest score (15 marks) for solutions of puzzles during the past month, the prize of £1 is divided equally between them, and the sum of 6*s*. 8*d*. will be forwarded to

Master OSWALD HIBBERT, Godley Vale, Godley, near Manchester (aged 10)

Master LESLIE ALLDRIDGE, 166, Rowland's Road, West Worthing.

Master W. R. MILLS, Colville House, Gratwicke Road, Worthing (aged 11).

*A. C. K.* has also sent in (15) correct solutions, but he is again disqualified as he has already received a prize since this competition began in October.

### Conditions.

I. Every paper sent in must be clearly written, and one side only must be used. All competitors must mark at the head of their papers the number of their competition.

II. Each paper must be signed by the author with his or her real name and address. A *nom de plume* may be added, if the writer does not desire to be referred to by us by his real name. In the case of all prize-winners, however, the real name and address will be published.

III. All communications relating to prize competitions should be addressed to "The Prize Editor, The Hospital, 38, Old Jewry, London, E.C." Competitors are requested not to include in letters, etc., to the Prize Editor communications on matters of other business. All letters must be fully prepaid.

IV. The Prize Editor of "The Hospital" is the sole judge of the competitions, and his decision is in all cases final and without appeal.

V. The Prize Editor reserves the right to publish any paper sent in, whether it receives a prize or not. He does not hold himself responsible for any MSS. sent, nor can he undertake to return rejected competitions.

**Notice to all Correspondents.**—N.B.—All letters referring to this page, which do not arrive at 38, Old Jewry, Cheapside, London, E.C., by the *first post on Thursdays*, and are not addressed PRIZE EDITOR, will in future be disqualified and disregarded.

No one will be permitted to win the Monthly or Quarterly Prizes twice in any one year, the Annual prizes being offered especially to prevent this.

